# How Incomputable Is Kolmogorov Complexity?

**DOI:** 10.3390/e22040408

**Published:** 2020-04-03

**Authors:** Paul M.B. Vitányi

**Affiliations:** 1 The National Research Center for Mathematics and Computer Science in the Netherlands (CWI), 1098XG Amsterdam, The Netherlands; paulv@cwi.nl; 2 Department of Computer Science, University of Amsterdam, 1012 WX Amsterdam, The Netherlands

**Keywords:** Kolmogorov complexity, incomputability, feasibility

## Abstract

Kolmogorov complexity is the length of the ultimately compressed version of a file (i.e., anything which can be put in a computer). Formally, it is the length of a shortest program from which the file can be reconstructed. We discuss the incomputability of Kolmogorov complexity, which formal loopholes this leaves us with, recent approaches to compute or approximate Kolmogorov complexity, which approaches are problematic, and which approaches are viable.

## 1. Introduction

Recently there have been several proposals regarding how to compute or approximate in some fashion the Kolmogorov complexity function. There is a proposal that is popular as a reference in papers that do not care about theoretical niceties, and a couple of proposals that do make sense but are not readily applicable. Therefore, it is timely to survey the field and show what is and what is not proven.

The plain Kolmogorov complexity was defined in [1] and denoted by *C* in the text [2] and its earlier editions. It deals with finite binary strings, *strings* for short. Other finite objects can be encoded into single strings in natural ways. The following notions and notation may not be familiar to the reader so we will briefly discuss them. The length of a string *x* is denoted by l(x). The *empty string* of 0 bits is denoted by ϵ. Thus, l(ϵ)=0. Let *x* be a natural number or finite binary string according to the correspondence

(ϵ,0),(0,1),(1,2),(00,3),(01,4),(10,5),(11,6),….

Then l(x)=⌊log(x+1)⌋. The Kolmogorov complexity C(x) of *x* is the length of a shortest string $x^{*}$ such that *x* can be computed from $x^{*}$ by a fixed universal Turing machine (of a special type called “optimal” to exclude undesirable such machines). In this way C(x) is a definite natural number associated with *x* and a lower bound on the length of a compressed version of it by any known or as yet unknown compression algorithm. We also use the conditional version C(x|y).

The papers by R.J. Solomonoff published in 1964, referenced as [3], contain informal suggestions about the incomputability of Kolmogorov complexity. Says Kolmogorov, “I came to similar conclusions [as Solomonoff], before becoming aware of Solomonoff’s work, in 1963–1964.” In his 1965 paper [1] Kolmogorov mentioned the incomputability of C(x) without giving a proof: “[…] the function $C_{\phi }\left(x|y\right)$ cannot be effectively calculated (generally computable) even if it is known to be finite for all *x* and *y*.” We give the formal proof of incomputability and discuss recent attempts to compute the Kolmogorov complexity partially, a popular but problematic proposal and some serious options. The problems of the popular proposal are discussed at length while the serious options are primarily restricted to brief citations explaining the methods gleaned from the introductions to the articles involved.

## 2. Incomputability

To find the shortest program (or rather its length) for a string *x* we can run all programs to see which one halts with output *x* and select the shortest. We need to consider only programs of length at most that of *x* plus a fixed constant. The problem with this process is known as the *halting problem* [4]: some programs do not halt, and it is undecidable which ones they are. A further complication is that we must show there are infinitely many such strings *x* for which C(x) is incomputable.

The first written proof of the incomputability of Kolmogorov complexity was perhaps in [5] and we reproduce it here following [2] to show what is and what is not proved.

**Theorem** **1.** *The function C(x) is not computable. Moreover, no partial computable function ϕ(x) defined on an infinite set of points can coincide with C(x) over the whole of its domain of definition.*

**Proof.**  We prove that there is no partial computable ϕ as in the statement of the theorem. Every infinite computably enumerable set contains an infinite computable subset, see e.g., [2]. Select an infinite computable subset *A* in the domain of definition of ϕ. The function ψ(m)=min{x:C(x)≥m,x∈A} is (total) computable (since C(x)=ϕ(x) on *A*), and takes arbitrarily large values, since it can obviously not be bounded for infinitely many *x*. Also, by definition of ψ, we have C(ψ(m))≥m. On the other hand, $C\left(\psi \left(m\right)\right)\leq C_{\psi }\left(\psi \left(m\right)\right)+c_{\psi }$ by definition of *C*, and obviously $C_{\psi }\left(\psi \left(m\right)\right)\leq l\left(m\right)$. Hence, m≤logm up to a constant independent of *m*, which is false from some *m* onward. □

That was the bad news; the good news is that we can approximate C(x).

**Theorem** **2.** *There is a total computable function ϕ(t,x), monotonic decreasing in t, such that $\lim_{t\to \infty }\phi \left(t,x\right)=C\left(x\right)$.*

**Proof.**  We define ϕ(t,x) as follows: For each *x*, we know that the shortest program for *x* has length at most l(x)+c with *c* a constant independent of *x*. Run the reference Turing machine *U* (an optimal universal one) for *t* steps on *each* program *p* of length at most l(x)+c. If for any such input *p* the computation halts with output *x*, then define the value of ϕ(t,x) as the length of the shortest such *p*, otherwise equal to l(x)+c. Clearly, ϕ(t,x) is computable, total, and monotonically nonincreasing with *t* (for all *x*, $\phi \left(t^{\prime },x\right)\leq \phi \left(t,x\right)$ if $t^{\prime }>t$). The limit exists, since for each *x* there exists a *t* such that *U* halts with output *x* after computing *t* steps starting with input *p* with l(p)=C(x). □

One cannot decide, given *x* and *t*, whether ϕ(t,x)=C(x). Since ϕ(t,x) is nondecreasing and goes to the limit C(x) for t→∞, if there were a decision procedure to test ϕ(t,x)=C(x), given *x* and *t*, then we could compute C(x). However, above we showed that *C* is not computable.

However, this computable approximation has no convergence guaranties as we show now. Let $g_{1},g_{2},\ldots$ be a sequence of functions. We call *f* the *limit* of this sequence if $f\left(x\right)=\lim_{t\to \infty }g_{t}\left(x\right)$ for all *x*. The limit is *computably uniform* if for every rational ϵ>0 there exists a t(ϵ), where *t* is a total computable function, such that $|f\left(x\right)-g_{t(\epsilon )}\left(x\right)|\leq \epsilon$, for all *x*. Let the sequence of one-argument functions $\psi_{1},\psi_{2},\ldots$ be defined by $\psi_{t}\left(x\right)=\phi \left(t,x\right)$, for each *t* for all *x*. Clearly, *C* is the limit of the sequence of ψs. However, by Theorem 1, the limit is not computably uniform. In fact, by the well-known halting problem, for each ϵ>0 and t>0 there exist infinitely many *x* such that $|C\left(x\right)-\psi_{t}\left(x\right)|>\epsilon$. This means that for each ϵ>0, for each *t* there are many *x*s such that our estimate ϕ(t,x) overestimates C(x) by an *error* of at least ϵ.

## 3. Computing the Kolmogorov Complexity

The incomputability of C(x) does not mean that we cannot compute C(x) for some *x*s. For example, if for individual string *x* we have C(C(x)|x)=c for some constant *c*, then this means that there is an algorithm of *c* bits which computes C(x) from *x*. We can express the incomputability of C(x) in terms of C(C(x)|x), which measures what we may call the “complexity of the complexity function.” Let l(x)=n. It is easy to prove the *upper bound* C(C(x)|x))≤logn+O(1). However, it is quite difficult to prove the lower bound [6]: For each length *n* there are strings *x* of length *n* such that

C(C(x)|x)≥logn−loglogn−O(1)

or its improvement by a game-based proof in [7]: For each length *n* there are strings *x* of length *n* such that

C(C(x)|x)≥logn−O(1).

This means that *x* only marginally helps to compute C(x); most information in C(x) is extra information related to the halting problem.

One way to go about computing the Kolmogorov complexity for a few small values is as follows. For example, let $T_{1},T_{2},\ldots$ be an acceptable enumeration of Turing machines. Such an acceptable enumeration is a formal concept ([2] Exercise 1.7.6). Suppose we have a fixed reference optimal universal Turing machine *U* in this enumeration. Let U(i,p) simulate $T_{i}\left(p\right)$ for all indexes *i* and (binary) programs *p*.

Run $T_{i}\left(p\right)$ for all *i* and *p* in the following manner. As long as *i* is sufficiently small it is likely that $T_{i}\left(p\right)<\infty$ for all *p* (the machine $T_{i}$ halts for every *p*). The Busy Beaver function BB(n):N→N was introduced in [8] and has as value the maximal running time of *n*-state Turing machines in quadruple format (see [8] or [2] for details). This function is incomputable and rises faster than any computable function of *n*.

Reference [9] supplies the maximal running time for halting machines for all i<5 and for i<5 it is decidable which machines halt. For i≥5 but still small there are heuristics [10,11,12,13]. A gigantic lower bound for all *i* is given in [14]. Using Turing machines and programs with outcome the target string *x* we can determine an upper bound on C(x) for reference machine *U* (by for each $T_{i}$ encoding *i* in self-delimiting format). Please note that there exists no computable lower bound function approximating C(x) since *C* is incomputable and upper semicomputable. Therefore it cannot be lower semicomputable [2].

For an approximation using small Turing machines we do not have to consider all programs. If *I* is the set of indexes of the Turing machines and *P* is the set of halting (or what we consider halting) programs then

$$\begin{array}{cc} {\left\{\left(i,p\right)\right\}}_{x}=\left\{\left(i,p\right):T_{i}\left(p\right)=x\right\}\backslash \{\left(i,p\right):T_{i}\left(p\right)=x & \wedge \exists_{i^{\prime },p^{\prime }}(T_{i^{\prime }}\left(p^{\prime }\right)=x\\ & \wedge |\left(i,p\right)|\leq \min\{|i^{\prime }|,|p^{\prime }|\}\}, \end{array}$$

with $i,i^{\prime }\in I,p,p^{\prime }\in P$. Here we can use the computably invertible Cantor pairing function [15] which is f:N×N→N defined by $f\left(a,b\right)=\frac{1}{2}\left(a+b\right)\left(a+b+1\right)+b$ so that each pair of natural numbers (a,b) is mapped to a natural number f(a,b) and vice versa. Since the Cantor pairing function is invertible, it must be one-to-one and onto: |(a,b)|=|a|+|b|. Here ${\left\{\left(i,p\right)\right\}}_{x}$ is the desired set of applicable halting programs computing *x*, i.e., if either $|i^{\prime }|$ or $|p^{\prime }|$ is greater than some |(i,p)| with $\left(i,p\right)\in {\left\{\left(i,p\right)\right\}}_{x}$ while $T_{i^{\prime }}\left(p^{\prime }\right)=x$ then we can discard the pair concerned from ${\left\{\left(i,p\right)\right\}}_{x}$.

## 4. Problematic Use of the Coding Theorem

Fix an optimal universal prefix Turing machine *U*. The *Universal distribution* (with respect to *U*) is $m\left(x\right)=\sum 2^{-l(p)}$ where *p* is a program (without input) for *U* that halts. The prefix complexity K(x) is with respect to the same machine *U*. The complexity K(x) is similar to C(x) but such that the set of strings for which the Turing machine concerned halts is prefix-free (no program is a proper prefix of any other program). This leads to a slightly larger complexity: K(x)≥C(x). The Coding theorem [16] states K(x)=−logm(x)+O(1). Since −logm(x)<K(x) (the term $2^{-K(x)}$ contributes to the sum and 2l(x)+O(logx) is also a program for *x*) we know that the O(1) term is greater than 0.

In [17] it was proposed to compute the Kolmogorov complexity by experimentally approximating the Universal distribution and using the Coding theorem. This idea was used in several articles and applications. One of the last is [18]. It contains errors or inaccuracies for example: “the shortest program” instead of “a shortest program,” “universal Turing machine” instead of “optimal universal Turing machine” and so on. Explanation: there can be more than one shortest program, and Turing machines can be universal in many ways. For instance, if U(p)=x for a universal Turing machine, the Turing machine $U^{\prime }$ such that $U^{\prime }\left(qq\right)=U\left(q\right)$ for every *q* and $U^{\prime }\left(r\right)=0$ for every string r≠qq for some string *q*, is also universal. Yet if *U* serves to define the Kolmogorov complexity C(x) then $U^{\prime }$ defines a complexity of *x* equal to 2C(x) which means that the invariance theorem does not hold for Universal Turing machines that are not optimal.

Let us assume that the computer used in the experiments fills the rôle of the required optimal Universal Turing machine for the desired Kolmogorov complexity, the target string, and the universal distribution involved. However, the O(1) term in the Coding theorem is mentioned but otherwise ignored in the experiments and conclusions about the value of the Kolmogorov complexity as reported in [17,18]. Yet the experiments only concern small values of the Kolmogorov complexity, say smaller than 20, so they are likely swamped by the constant hidden in the O(1) term. Let us expand on this issue briefly. In the proof of the Coding theorem, see e.g., [2], a Turing machine *T* is used to decode a complicated code. The machine *T* is one of an acceptable enumeration $T_{1},T_{2},\ldots$ of all Turing machines. The target Kolmogorov complexity *K* is shown to be smaller than the complexity $K_{T}$ associated with *T* plus a constant *c* representing the number of bits to represent *T* and other items: $K\left(x\right)\leq K_{T}\left(x\right)+c$. Since *T* is complex since it serves to decode this code, the constant *c* is huge, i.e., much larger than, say, 100 bits. The values of *x* for which K(x) is approximated by [17,18] are at most 5 bits, i.e., at most 32. Unless there arises a way to prove the Coding theorem without the large constant *c*, this method does not seem to work. Other problems: The distribution m(x) is apparently used as $m\left(x\right)=\sum_{i\in N,T_{i}\left(\epsilon \right)=x}2^{-l(\epsilon )}/i$, see ([19] Equation (6)) using a (noncomputable) enumeration of Turing machines $T_{1},T_{2},\ldots$ that halt on empty input ϵ. Therefore $\sum_{x\in N}m\left(x\right)=\sum_{i\in N,T_{i}\left(\epsilon \right)<\infty }2^{-l(\epsilon )}/i$ and with l(ϵ)=0 we have $\sum_{x\in N}m\left(x\right)=\infty$ since $\sum_{x\in N}1/x=\infty$. By definition however $\sum_{x\in N}m\left(x\right)\leq 1$: contradiction. It should be $m\left(x\right)=\sum_{i\in N,T_{i}\left(p\right)=x}2^{-l(p)-\alpha (i)}$ with $\sum_{i\in N}\alpha \left(i\right)\leq 1$ as shown in ([2] pp. 270–271).

## 5. Natural Data

The Kolmogorov complexity of a file is a lower bound on the length of the ultimate compressed version of that file. We can approximate the Kolmogorov complexities involved by a real-world compressor. Since the Kolmogorov complexity is incomputable, in the approximation we never know how close we are to it. However, we assume in [20] that the natural data we are dealing with contain no complicated mathematical constructs like π=3.1415… or Universal Turing machines, see [21]. In fact, we assume that the natural data we are dealing with contains primarily effective regularities that a good compressor finds. Under those assumptions the Kolmogorov complexity of the object is not much smaller than the length of the compressed version of the object.

## 6. Safe Computations

A formal analysis of the intuitive idea in Section 5 was subsequently and independently given in [22]. From the abstract of [22]: “Kolmogorov complexity is an incomputable function. *…* By restricting the source of the data to a specific model class, we can construct a computable function to approximate it in a probabilistic sense: the probability that the error is greater than *k* decays exponentially with *k*.” This analysis is carried out but its application yielding concrete model classes is not.

## 7. Short Lists

Quoting from [23]: “Given that the Kolmogorov complexity is not computable, it is natural to ask if given a string *x* it is possible to construct a short list containing a minimal (plus possibly a small overhead) description of *x*. Bauwens, Mahklin, Vereshchagin and Zimand [24] and Teutsch [25] show that surprisingly, the answer is YES. Even more, in fact the short list can be computed in polynomial time. More precisely, the first reference showed that one can effectively compute lists of quadratic size guaranteed to contain a description of *x* whose size is additively O(1) from a minimal one (it is also shown that it is impossible to have such lists shorter than quadratic), and that one can compute in polynomial-time lists guaranteed to contain a description that is additively O(logn) from minimal. Finally, Ref. [25] improved the latter result by reducing O(logn) to O(1)”. See also [26].

## 8. Conclusions

The review shows that the Kolmogorov complexity of a string is incomputable in general, but may be computable for some arguments. To compute or approximate the Kolmogorov complexity, several approaches have recently been proposed. The most popular of these is inspired by L.A. Levin’s Coding theorem and consists of taking the negative logarithm of the so-called universal probability of the string to obtain the Kolmogorov complexity of very short strings (this is not excluded by incomputability as we saw). This probability is approximated by the frequency distributions obtained from small Turing machines. As currently stated, the approach is problematic in the sense that it is only suggestive and cannot be proved correct. Nonetheless, some applications make use of it. Proper approaches either restrict the domain of strings of which the Kolmogorov complexity is desired (so that the incomputability turns into computability) or manage to restrict the Kolmogorov complexity of a string to an item in a small list of options (so that the Kolmogorov complexity has a certain finite probability).
